# Rice Overexpressing *OsNUC1-S* Reveals Differential Gene Expression Leading to Yield Loss Reduction after Salt Stress at the Booting Stage

**DOI:** 10.3390/ijms19123936

**Published:** 2018-12-07

**Authors:** Chuthamas Boonchai, Thanikarn Udomchalothorn, Siriporn Sripinyowanich, Luca Comai, Teerapong Buaboocha, Supachitra Chadchawan

**Affiliations:** 1 Center of Excellence in Environment and Plant Physiology, Department of Botany, Faculty of Science, Chulalongkorn University, Bangkok 10330, Thailand; Gwang_desu@hotmail.com (C.B.); Thanikarn@sut.ac.th (T.U.); 2 Surawiwat School, Suranaree University of Technology, Nakhon Ratchasima 30000, Thailand; 3 Faculty of Liberal Arts and Science, Kasetsart University, Kamphaeng Saen Campus, Nakhon Pathom 73140, Thailand; wanich_s@hotmail.com; 4 Department of Plant Biology and Genome Center, University of California Davis, Davis, CA 95616, USA; lcomai@ucdavis.edu; 5 Department of Biochemistry, Faculty of Science, Chulalongkorn University, Bangkok 10330, Thailand; Teerapong.B@chula.ac.th; 6 Omics Science Center, Faculty of Science, Chulalongkorn University, Bangkok 10330, Thailand

**Keywords:** RNA binding protein, nucleolin, salt stress, photosynthesis, light saturation point, booting stage, transcriptome

## Abstract

Rice nucleolin (OsNUC1), consisting of two isoforms, OsNUC1-L and OsNUC1-S, is a multifunctional protein involved in salt-stress tolerance. Here, *OsNUC1-S*’s function was investigated using transgenic rice lines overexpressing *OsNUC1-S*. Under non-stress conditions, the transgenic lines showed a lower yield, but higher net photosynthesis rates, stomatal conductance, and transpiration rates than wild type only in the second leaves, while in the flag leaves, these parameters were similar among the lines. However, under salt-stress conditions at the booting stage, the higher yields in transgenic lines were detected. Moreover, the gas exchange parameters of the transgenic lines were higher in both flag and second leaves, suggesting a role for *OsNUC1-S* overexpression in photosynthesis adaptation under salt-stress conditions. Moreover, the overexpression lines could maintain light-saturation points under salt-stress conditions, while a decrease in the light-saturation point owing to salt stress was found in wild type. Based on a transcriptome comparison between wild type and a transgenic line, after 3 and 9 days of salt stress, the significantly differentially expressed genes were enriched in the metabolic process of nucleic acid and macromolecule, photosynthesis, water transport, and cellular homeostasis processes, leading to the better performance of photosynthetic processes under salt-stress conditions at the booting stage.

## 1. Introduction

Rice (*Oryza sativa* L.) is a staple food and a main source of energy for humans, especially in Asia. There are several biotic or abiotic stresses that limit rice growth and yield, such as soil salinity, drought, and soil nutrition [1]. Salinity is a severe abiotic stress worldwide that directly contributes to the economic outcome of agriculturists. It negatively affects plants at both physiological and cellular levels. The plant water absorption is disrupted, leading to reductions in plant growth and development [2]. Moreover, ion toxicity also causes changes in plant metabolism, including the photosynthesis processes and energy production [3]. It directly affects photosynthetic components, including chlorophyll *a*, chlorophyll *b*, and carotenoids, because salt stress increases enzyme activities involved in chlorophyll degradation, which leads to a decrease in chlorophyll levels [4]. Moreover, it can also induce reactive oxygen species (ROS) production, which can trigger protein and lipid damage [5]. The level of plant injury depends on species, developmental stage, age, and also the severity of the salinity.

Rice is strongly affected by salt stress at both the seedling and reproductive stages [6]. These can remarkably affect rice plants that are grown in the paddy field of rain-fed areas containing rock salt underneath, like the northeastern region of Thailand [7], because rice is normally germinated at the beginning of the rainy season, when the salinity is low due to a certain amount of rain. However, when the land is drying from the evaporation due to the lack of rain, the salinity can migrate from the rock salt underneath through the surface [7], causing salt stress at the certain developmental stage of rice. If this occurs at the reproductive stage, it becomes the major effect for plant yield reduction [8].

Nucleolin, a multifunctional protein, is localized in various cellular locations, including the nucleus. It consists of three domains, the N-terminus, containing several acidic stretches, the central region, containing an RNA-binding domain, and the C-terminus, containing a glycine/arginine-rich domain [9]. Because of differences in the numbers of each motif, different functions were found in different species. For example, the *NUC-L1* gene in *Arabidopsis thaliana* is related to its growth and development [10], whereas in pea, this gene is regulated by light [11].

In rice, two forms of Nucleolin1 were found, a longer (*OsNUC1-L*, GenBank Accession No. AK103446) and a shorter (*OsNUC1-S*, GenBank Accession No. AK063918) form. *OsNUC1-S* lacks an N-terminal region, but the cDNA sequences of the other regions are the same as the longer form. In 2013, Sripinyowanich and colleagues studied *OsNUC1-S* overexpression in *Arabidopsis* and found that this form promoted salt tolerance by increasing the number of lateral roots and enhancing root growth. In addition, the overexpression of this gene in rice leads to shoot fresh weight increasing when seedlings are grown under salt-stress conditions [12]. In the reproductive stage, the function of this gene is still unknown. Recently, the overexpression of the *OsNUC1-L* form resulted in an increase in salt tolerance in both rice and *Arabidopsis* by enhancing photosynthesis [13].

Here, we investigate the effects of *OsNUC1-S* overexpression on photosynthetic responses under normal and salt-stress conditions in transgenic rice at the reproductive stage and use a transcriptome analysis to explore the gene expression levels affected by *OsNUC1-S* overexpression. The experiments were performed in flag leaves and second leaves as both leaves have been reported to have a major role in generating carbohydrate resource from the photosynthesis process for seed production [14].

## 2. Results

### 2.1. Overexpression of OSNUC1-S Affects Rice Yield

**Figure 1:**
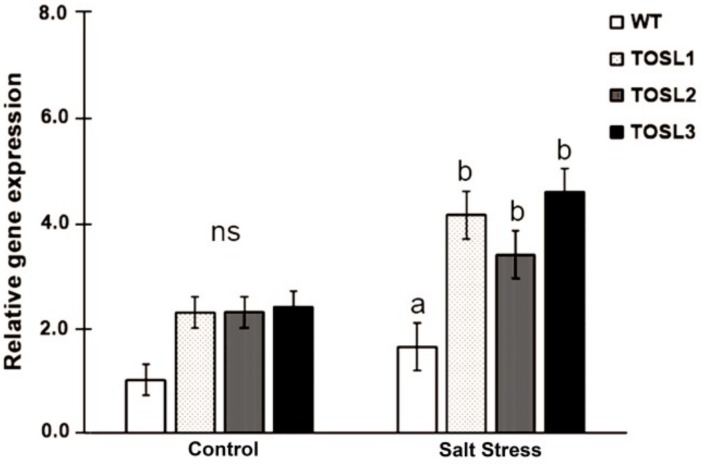
*OsNUC1* gene expression in wild type (WT) and *OsNUC1-S* over-expression lines, TOSL1, TOSL2, and TOSL3, in the control and salt stress condition. Analysis of variance was performed and means were compared with Tukey’s range test analysis. The data were presented as the mean ± SE and a different letter above the bar showed the significant difference in means (*p* < 0.05). ns represents no statistically difference among means.

The transgenic rice lines, TOSL1, TOSL2, and TOSL3, with *OsNUC1-S* overexpression were used in these experiments. *OsNUC1* expression was compared among wild type (WT) and the transgenic lines, when grown in the control and salt stress condition at reproductive stage. Significantly higher *OsNUC1* gene expression could be detected in transgenic lines when compared to WT, especially in the salt stress condition (Figure 1).

To investigate if the overexpression of *OsNUC1-S* affected rice productivity, the tiller number per plant, panicle number per plant, panicle length, fertility rate (%), and seed number per plant of the transgenic rice, TOSL1, TOSL2, TOSL3, and WT, were evaluated as shown in Table 1. In the normal grown condition (control), over-expression of *OsNUC1-S* caused the reduction in % fertility, leading to the reduction in seed number per plant. However, under salt stress, the transgenic lines tended to have the higher tiller number per plant, panicle number per plant, panicle length, % fertility, and seed number per plant, except the TOSL2 line that showed similar % fertility and seed number per plant to WT. The reduction percentage of seeds/plants in WT was 64%, while the transgenic lines had the seeds/plant reduction of 17%, 47%, and 27%. These data suggested that there should be some changes in metabolisms in the transgenic lines due to *OsNUC1-S* over-expression.

### 2.2. Overexpression of OSNUC1-S Increased the Photosynthetic Rate, Stomatal Conductance, and Transpiration Rate under Salt-Stress Conditions

**Figure 2:**
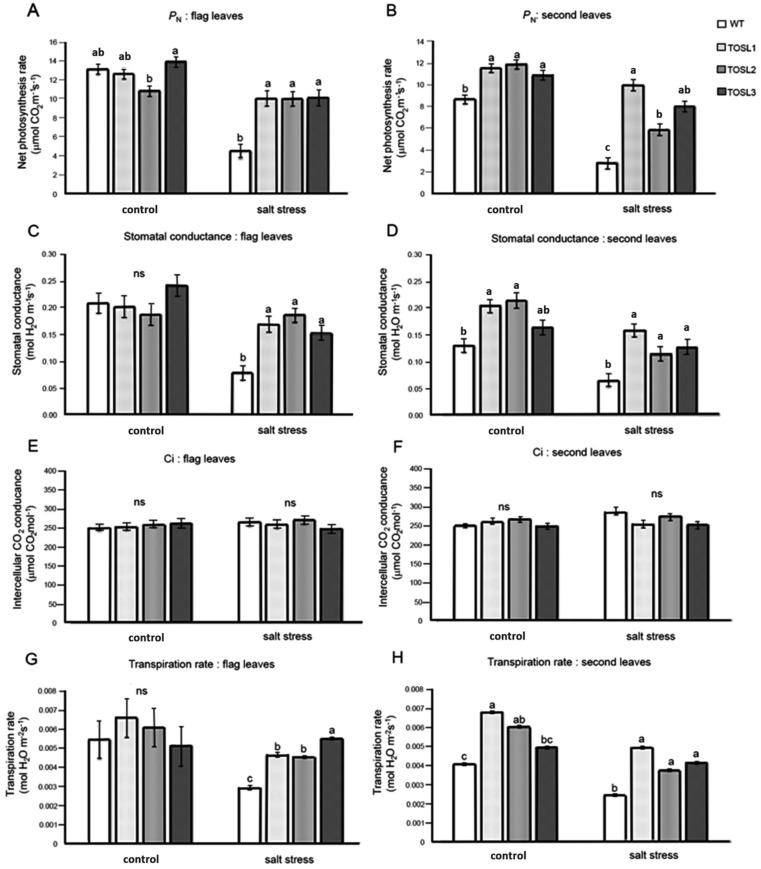
The net photosynthetic rate (P_N_) (**A**,**B**), stomatal conductance (g_s_) (**C**,**D**), intercellular CO_2_ concentration (Ci) (**E**,**F**), and transpiration rate (*E*) (**G**,**H**) of flag leaves (**A**,**C**,**E**,**G**) and second leaves (**B**,**D**,**F**,**H**), when wild type (WT) and the *OsNUC1–S* overexpressing lines, TOSL1, TOSL2, and TOSL3, were grown under control or salt-stress conditions. The data were presented as the mean ± SE and a different letter above the bar showed the significant difference in means (*p* < 0.05) based on Tukey’s range test analysis. ns represents no statistically difference among means.

Salt stress can cause a decrease in productivity in rice, and the major organs generating the carbohydrate for grain filling are flag leaves and second leaves. Therefore, we investigated the photosynthetic activities in both of these leaves in WT and *OsNUC1-S* over-expressing lines under control and salt stress conditions. Under the control condition, only second leaves of transgenic rice overexpressing *OSNUC1-S* showed higher net photosynthetic rates (P_N_) and stomatal conductance levels (g_s_), while the flag leaves had similar levels, except TOSL2, which had a lower P_N_ than the other lines. These results may reflect positional effects of the transformation. After 9 days of salt stress, the overexpression of *OsNUC1-S* increased the P_N_ and g_s_ of all the transgenic lines (Figure 2). The P_N_ values in flag leaves of transgenic plants were approximately two-fold those of the WT (Figure 2A), while in second leaves, the P_N_ values of transgenic plants increased up to 2.5-fold those of WT (Figure 2B). Similar effects were also found for the g_s_ values of both flag and second leaves (Figure 2C,D). There was no effect on the intercellular CO_2_ concentration (Ci) (Figure 2E,F), but *OsNUC1-S*’s overexpression resulted in an increased transpiration rate of the second leaves under control conditions and in the flag leaves under salt-stress conditions (Figure 2G,H).

### 2.3. Overexpression of OSNUC1-S Affected Both Light-Response Curves and CO_2_-Response Curves of Flag and Second Leaves under Salt-Stress Conditions

**Figure 3:**
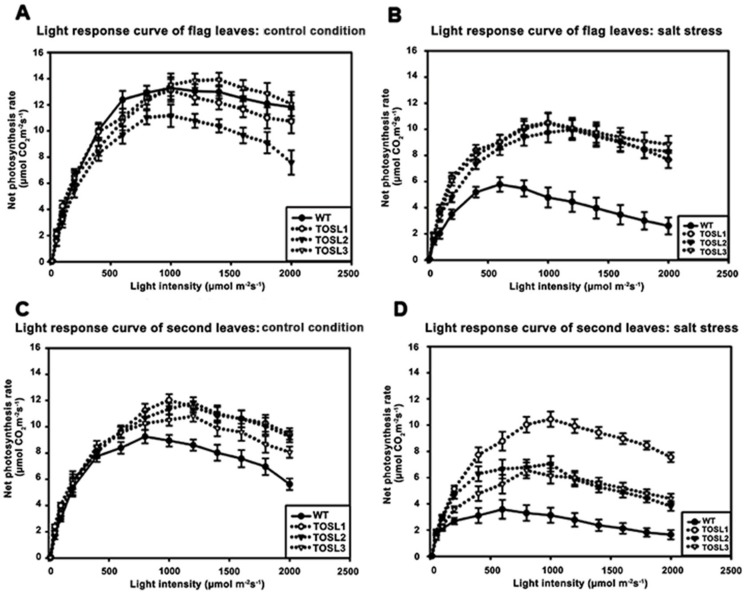
Light-response curves of flag leaves (**A**,**B**) and second leaves (**C**,**D**), when wild type and transgenic lines, TOSL1, TOSL2, and TOSL3, were grown under control (**A**,**C**) and salt-stress (**B**,**D**) conditions.

We investigated the light-response curves of these lines. The light-response curves of the flag leaves in all the lines were similar when the light intensity varied from 100 to 2000 µmol·m^−2^·s^−1^, except TOSL2, which had a lower P_N_ than other lines. The light-saturation points were approximately 1000 µmol·m^−2^·s^−1^ in all lines (Figure 3A). The salt stress caused decreases in the P_N_ values of all the lines, but the transgenic lines had significantly higher P_N_ values when the light intensity was greater than 200 µmol·m^−2^·s^−1^. The light-saturation point of the WT decreased to 600 µmol·m^−2^·s^−1^, while the transgenic lines had light-saturation points similar to those of plants grown under control conditions (1000 µmol·m^−2^·s^−1^) (Figure 3B).

The second leaves of the WT had lower light-saturation points than those of the transgenic lines when grown under control conditions. The P_N_ values of the WT’s second leaves started to decline when the light intensity was greater than 800 µmol·m^−2^·s^−1^; however, for the transgenic lines, the light-saturation point was 1000 µmol·m^−2^·s^−1^ (Figure 3C).

Salt stress caused a decrease in the light-saturation point in WT second leaves, but it did not affect the light-saturation points of the transgenic lines’ second leaves. In WT second leaves, the light-saturation point declined to 600 µmol·m^−2^·s^−1^, and a more than two-fold reduction in the P_N_ was found. A similar reduction in the P_N_ was found in the transgenic lines, except TOSL1, which maintained a P_N_ in its second leaves that was similar to the P_N_ under control growth conditions. However, all the transgenic lines’ second leaves had light-saturation points of 1000 µmol·m^−2^·s^−1^ (Figure 3D).

**Figure 4:**
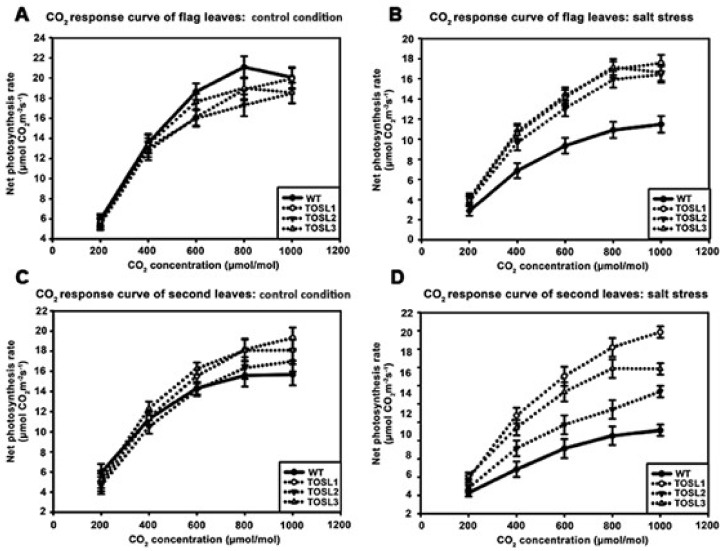
CO_2_-response curves of flag leaves (**A**,**B**) and second leaves (**C**,**D**), when wild type and transgenic lines, TOSL1, TOSL2, and TOSL3, were grown under control (**A**,**C**) and salt-stress (**B**,**D**) conditions.

The CO_2_-response curves of flag and second leaves of all the lines were also investigated as the CO_2_ concentration increased from 200 to 1000 µmol·mol^−1^. Under control conditions, the flag leaves of all the lines showed similar CO_2_-response curves. The CO_2_-saturation point was ~800 µmol·mol^−1^ (Figure 4A). Under salt-stress conditions, significantly higher P_N_ values of the flag leaves were found in all the transgenic lines when the CO_2_ concentration was greater than 200 µmol·mol^−1^. However, salt stress had no effect on the CO_2_-saturation points of flag leaves in any line (Figure 4B).

In the second leaves, the CO_2_-response curves and CO_2_-saturation points of all the lines were similar and consistent with the flag leaf’s response to the lower P_N_ in plants grown under control conditions (Figure 4C). Salt stress caused decreases in the P_N_ values of all the lines, but it did not affect the CO_2_ saturation point of WT second leaves. On the contrary, TOSL1 and TOSL2’s second leaves had increased CO_2_ saturation points to over 1000 mmol·mol^−1^, while TOSL3 showed the same CO_2_ saturation point as WT second leaves, when grown under salt-stress conditions (Figure 4D).

### 2.4. Salt Stress Affects Photosystem II (PSII) Photochemistry Efficiency and Photosynthetic Pigment Contents in Flag and Second Leaves

**Figure 5:**
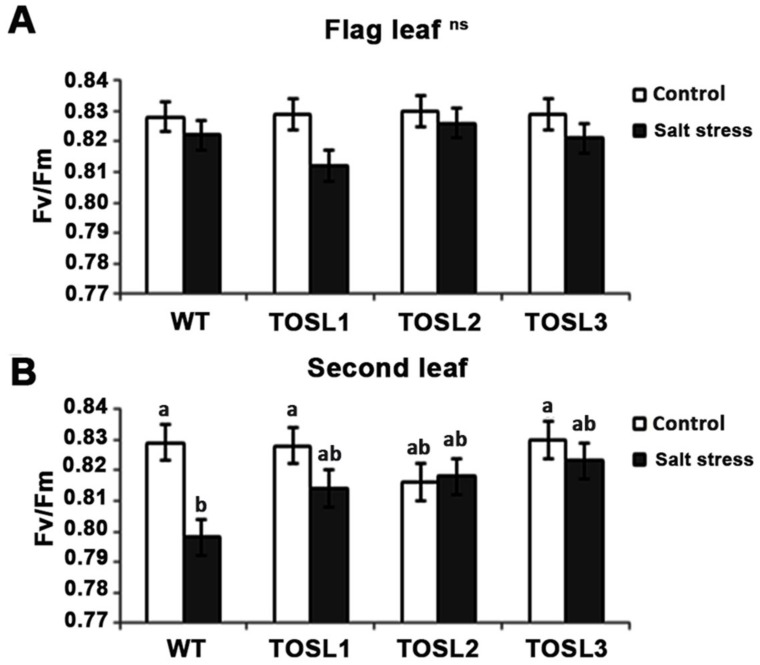
The F_v_/F_m_ ratios of flag leaves (**A**) and second leaves (**B**) under control conditions and after being treated with 150 mM NaCl solution for 9 days. Three independent *OsNUC1-S* transgenic rice lines with difference transgene expression levels and WT plants were used. The data were presented as the mean ± SE and a different letter above the bar showed the significant difference in means (*p* < 0.05) based on Tukey’s range test analysis. ns represents no statistically difference among means.

To investigate the effect of salt stress on the efficiency of PSII photochemistry, the F_v_/F_m_ ratio was investigated. The salt-stress level did not significantly affect the PSII efficiency in the flag leaves of any lines (Figure 5A). However, in the second leaves, a significant reduction in the PSII efficiency (F_v_/F_m_) was found in WT after 9 days of salt treatment, while in all the transgenic lines, a reduced effect was found (Figure 5B). Thus, second leaves were more susceptible to salt stress than flag leaves, and *OsNUC1-S*’s overexpression contributed to the PSII photochemistry efficiency under salt-stress conditions.

**Figure 6:**
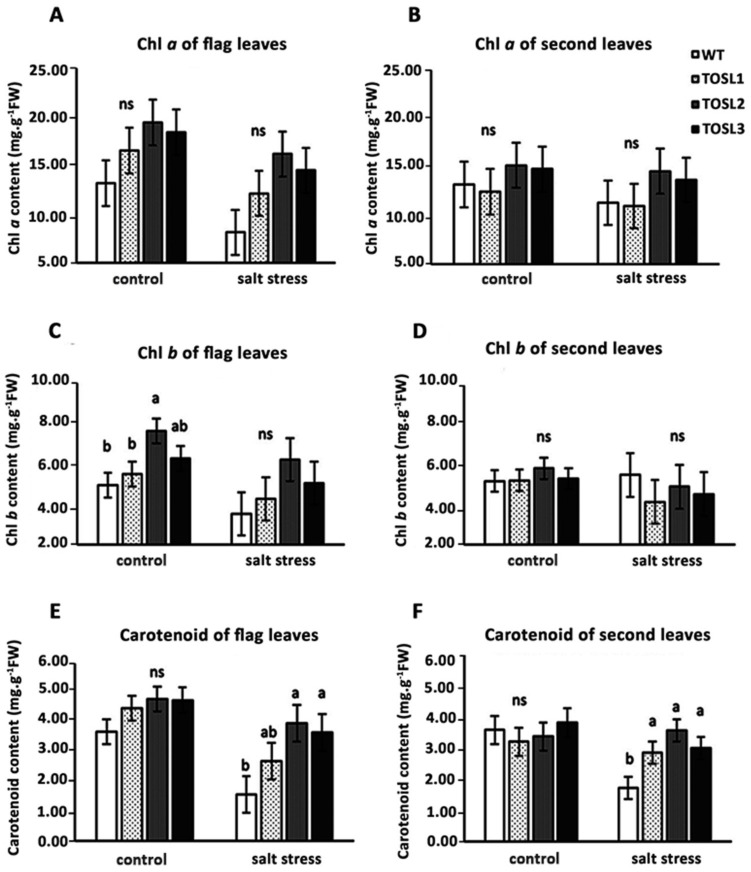
Photosynthetic pigments in flag leaves (**A**,**C**,**E**) and second leaves (**B**,**D**,**F**) 9 days after the salt-stress treatment at the booting stage. The data were presented as the mean ± SE and a different letter above the bar showed the significant difference in means (*p* < 0.05) based on Tukey’s range test analysis. ns represents no statistically significant difference among means.

*OsNUC1-S*’s overexpression tended to increase both the chlorophyll and carotenoid contents in flag leaves, but not in the second leaves, under optimal growth conditions (Figure 6A–F). Salt stress caused decreases in all of the pigments in the flag leaves, but significantly higher carotenoid contents were found in both flag and second leaves in the transgenic lines when compared with WT (Figure 6E,F).

### 2.5. OsNUC1-S’s Overexpression Increased Carbohydrate Metabolism and Sugar Transport in Flag Leaves of Rice Grown Under Control Conditions

Because of the effects of *OsNUC1-S*’s overexpression on the photosynthetic characteristics, the transcriptome approach was used to investigate changes at the transcript level. TOSL3 was chosen as the representative for investigations of the flag leaf transcriptome. Based on a transcriptome comparison of DEGs (differentially expressed genes) between WT and TOSL3, when grown under control conditions, the DEGs were highly enriched in the cellular macromolecule metabolic processes, including the macromolecule biosynthetic process. Genes involved in transmembrane transport, regulation of cellular process, and the pigment metabolic process were also found.

**Figure 7:**
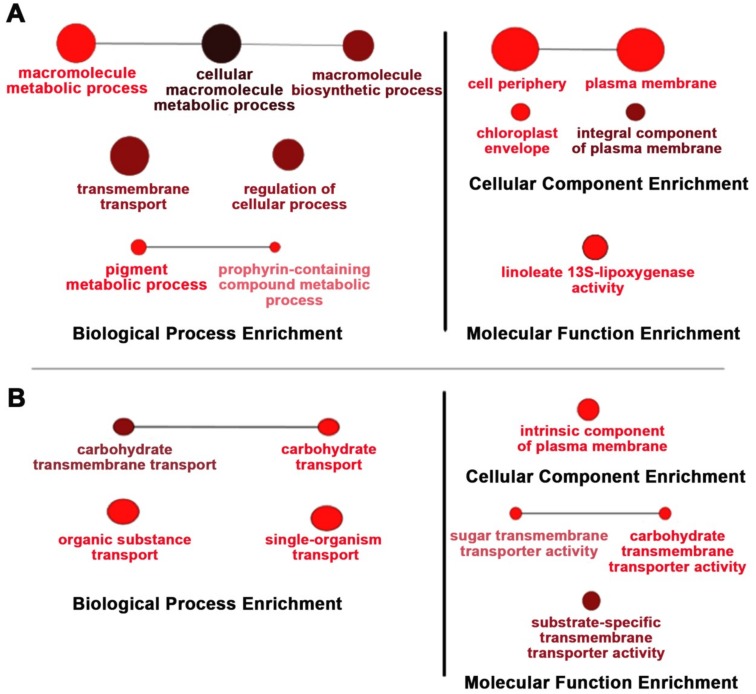
Gene enrichment analysis of the differentially expressed genes in the TOSL3 line, which overexpresses *OsNUC1-S*. The tissues for the transcriptome analysis were collected 3 days (**A**) and 9 days (**B**) after the first day of the booting stage. The darker colors represent the higher significance and the larger size of node represents the higher number of genes in the group.

For the cellular component enrichment, a plasma membrane and chloroplast envelope were reported. This supported *OsNUC1-S*’s role in the enhancement of macromolecule production for grain filling in the flag leaves. Interestingly, the molecular function enrichment was found for only linoleate 13S-lipoxygenase activity. This enzyme is involved in plant growth and development [15], and also in the wounding response through jasmonic acid (JA) signaling [16,17] (Figure 7A).

Six days later, the DEGs resulting from *OsNUC1-S* expression changed. For the biological process, the genes functioning in carbohydrate transport were enriched, supporting the role of flag leaves in seed development. The consistent enrichment of molecular function in substrate-specific transmembrane transport was found (Figure 7B).

### 2.6. The Overexpression of OsNUC1-S Increased the Expression of Genes Involved in Water Transport and Cellular Homeostasis, Nucleic Acid and Macromolecule Metabolic Processes, and Photosynthetic Processes

**Figure 8:**
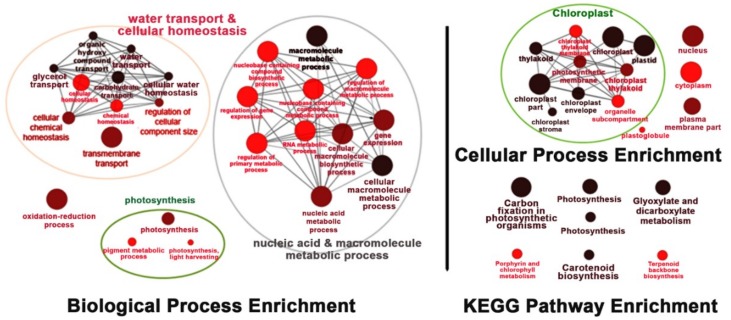
The enrichment for the biological process, cellular process, and Kyoto Encyclopedia of Genes and Genomes (KEGG) pathways of the differentially expressed genes in the flag leaves of transgenic rice overexpressing *OsNUC1-S* after 3 days of salt stress. The darker colors represent the higher significance and the larger size of node represents the higher number of genes in the group.

Under salt-stress conditions, the effects of *OsNUC1-S* overexpression were different from the effects found in normally grown plants. After 3 days of salt stress, the flag leaves of the transgenic lines were enriched with transcripts of genes involved in water transport and cellular homeostasis, nucleic and macromolecule metabolic processes, and photosynthetic processes. This was also consistent with the cellular enrichment in chloroplasts and nuclei. For the KEGG pathways, enrichment occurred in photosynthesis, carbon fixation, porphyrin and chlorophyll metabolism, and carotenoid biosynthesis, all of which involved activities in chloroplasts. Moreover, genes in glyoxylate and dicarboxylate metabolism, and terpenoid backbone biosynthesis, were also enriched (Figure 8).

**Figure 9:**
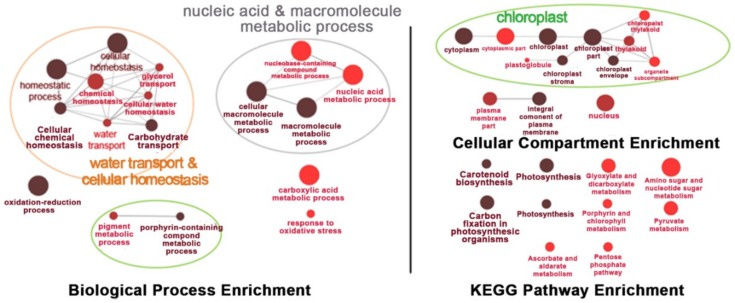
The enrichment for biological process, cellular process, and KEGG pathways of the differentially expressed genes in the flag leaves of transgenic rice overexpressing *OsNUC1-S* after 9 days of salt stress. The darker colors represent the higher significance and the larger size of the node represents the higher number of genes in the group.

When the plants were under a prolonged stress for 9 days, similar processes, cellular compartments, and metabolic pathways were found to be affected, except that pigment and porphyrin-containing compound metabolic processes were detected, suggesting more pigment synthesis-related processes occurred, while the enrichment in light harvesting was not found. A new set of enriched genes found at this time point was in carboxylic acid metabolic processes and responses to oxidative stress. Chloroplasts were the main organelles that were enriched with the transcripts of genes affected by *OsNUC1-S* expression. The enrichment of the genes in the nucleus and plasma membrane also had a similar pattern to that found in flag leaves after 3 days of salt stress. The pathway enrichment after salt stress for 9 days was similar to that found after 3 days of stress, with more pathways in amino sugar and nucleotide sugar metabolism, pyruvate metabolism, ascorbate and aldarate metabolism, and pentose phosphate pathways (Figure 9). The list of the differentially expressed genes between wild type and transgenic rice with *OsNUC1-S* overexpression after salt stress treatment for 3 and 9 days is shown in Supplementary Table S1.

**Figure 10:**
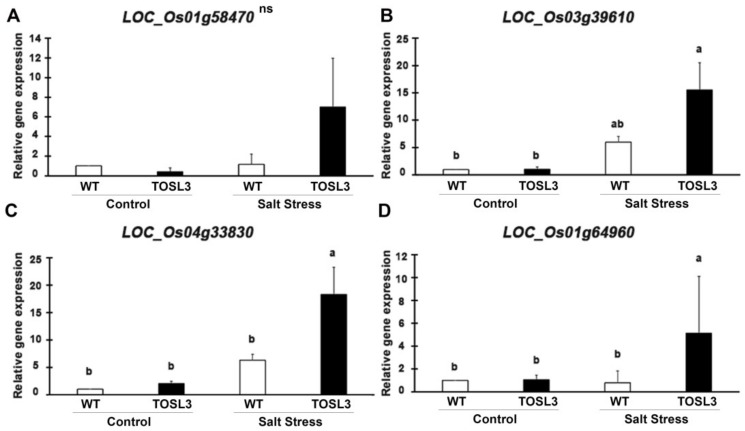
Gene expression analysis of *LOC_Os01g58470* (**A**), *LOC_Os03g39610* (**B**), *LOC_Os04g33830* (**C**), and *LOC_Os01g64960* (**D**) in WT and TOSL3 plants under control and salt stress conditions. Bar represents standard deviation of three biological replicates. The measurement was performed with three technical replicates. The data were presented as the mean ± SE and a different letter above the bar showed the significant difference in means (*p* < 0.05) based on Tukey’s range test analysis. ns represents no statistically significant difference among means.

To validate the RNA-Seq data, the up-regulated genes after 3 days of salt stress, *LOC_Os01g58470* (*CEST*) (Figure 10A), *LOC_Os03g39610* (*CAB*) (Figure 10B), and *LOC_Os04g33830* (*PSAO*) (Figure 10C), were chosen as representatives for quantitative RT-PCR (qRT-PCR). The expression in the transgenic line, TOSL3 was about two- to three-fold higher than WT under salt stress condition, based on RNA-Seq analysis (Supplementary Table S1). The comparable expression was also detected by qRT-PCR as shown in Figure 10A–C. Based on RNA-Seq analysis, *LOC_Os01g64960* (*PsbS1*) (Figure 10D) showed the highest fold change when compared to WT after salt stress for 9 days. The qRT-PCR of this gene expression revealed about five-fold higher expression than WT after 9 days (Figure 10D). The increase in the expression of these genes was correlated with the RNA-Seq data.

## 3. Discussion

*OsNUC1-S*, one of the mRNA splice forms, lacks an N-terminal region that contains several acidic stretches and a nuclear localization signal. However, based on the localization study [10], the protein localizes to both the cytoplasm and nucleus. The overexpression of *OsNUC1-S* did not change the photosynthetic activity levels in flag leaves when plants were grown under control conditions (Figure 2), but it resulted in reduced rice seed numbers and fertility rates (Table 1). The transcriptomic analysis found increased levels of carbohydrate metabolism and sugar transmembrane transport (Figure 7). This was in agreement with the role of flag leaves as the energy source for rice grain development [18]. Interestingly, TOSL3 was enriched in linoleate 13S-lipoxygenase activity (Figure 7). Lipoxygenase is the enzyme involved in JA biosynthesis and in responding to wounding and stress [17]. The exogenous application of JA induces flag leaf senescence by regulating chlorophyll degradation, membrane deterioration, and SAG (senescence associated gene) expression levels [19]. Moreover, the overexpression of the *TIFY* gene could enhance the rice grain yield, possibly owing to a reduction in JA sensitivity [20]. Therefore, the increase in lipoxygenase activity in the *OsNUC1-S* overexpression line could increase the JA level, causing decreases in the rice grain yield and fertility rate (Table 1). Both flag and second leaves contribute to the grain-filling process [14], but flag leaves provide more than 50% of the assimilates for grain filling [21]. In a comparison between WT and transgenic lines, a greater P_N_ was found only in the second leaves of the transgenic lines when grown under salt-stress conditions. This may be related to the maximization of the photosynthetic capacity of the flag leaves.

Under salt stress, a threefold reduction of P_N_ was detected in WT, while only a 10–15% reduction was found in the flag leaves of transgenic lines (Figure 2A). This suggested a role for *OsNUC1-S* in photosynthetic enhancement. The increase in photosynthetic activity was supported by the enrichment of genes involved in the photosynthetic processes, as well as in water transport and cellular homeostasis activities (Figure 8). The enrichment in nucleic acid and macromolecule metabolic processes supports a role for nucleolin that involves RNA modifications. Thus, OsNUC1-S may have a specific target for its activity, which results in greater changes in the expression levels of some genes compared with others when *OsNUC1-S* is overexpressed. The enrichment in carbon fixation, as well as porphyrin and chlorophyll metabolism, suggests that *OsNUC1-S* enhanced both the light reaction and the carbon fixation process. The increased light-saturation point (Figure 3) and carbon fixation activity (Figure 4) were consistent with the transcriptome data. Interestingly, when plants were impacted by salt, the function of *OsNUC1-S* changed. Both the transcriptome and physiological data indicated that this gene promoted salt tolerance through enhanced photosynthesis. Genes encoding proteins in the photosynthetic processes were strongly expressed in transgenic lines compared with WT during salt stress (Supplementary Table S1, Figure 10). Most of the up-regulated genes were located in PSI and PSII of the light reaction. Moreover, some genes in the Calvin cycle also increased, possibly causing the higher P_N_ values in transgenic plants. The enriched genes encoding chlorophyll A-B-binding protein (Supplementary Table S1), which functions in light harvesting, were also up-regulated in transgenic plants. This supported the light-response curve results in which a higher light-saturation point occurred in transgenic plants under salt stress.

The second leaves in WT were more susceptible to salt stress than flag leaves. The former had a significant decrease in F_v_/F_m_ after 9 days of salt stress, while salt stress had no effect on flag leaves. Na^+^ can be transferred from root to shoot, and in rice, *OsHKT1;5* excludes Na^+^ from the phloem to prevent Na^+^ transfer to the younger leaf blades [22]. Therefore, the second leaves were affected by salt stress to a larger extent than the flag leaves. This may be the mechanism that prevents Na^+^ toxicity from reaching the flag leaf, which has the main role in carbon fixation for grain development.

## 4. Materials and Methods

### 4.1. Plant Material and Salt Treatment

The experiment was conducted in a planting house for transgenic plants in the Botany Department, Faculty of Science, Chulalongkorn University in Thailand. Three independent transgenic rice lines expressing *OsNUC1-S* driven by the Ubiquitin promoter have been produced by Sripinyowanich and colleagues [12] in a “Nipponbare” rice genetic background, and the homozygous T_3_ generations of these transgenic lines were used in this experiment. “Nipponbare” rice seeds were obtained from the National Laboratory for Protein Engineering and Plant Genetic Engineering, Peking-Yale Joint Research Center for Plant Molecular Genetics and AgroBiotechnology, Peking University, People’s Republic of China. Either wild type (WT) or transgenic seeds were germinated for 7 days. Then, seedlings were transferred to pots containing 5 kg of soil. When plants were at the booting stage, 150 mM NaCl solution was added to cause salt stress at 8–10 ds·m^−1^. All photosynthetic parameters were measured after 9 days salt treatment using a Gas Analysis System (LI-COR, LI-6400, USA) with a 1200 µmol·m^−2^·s^−1^ light intensity and 380 µmol mol^−1^ carbon dioxide (CO_2_) concentration.

For chlorophyll fluorescence parameters, either the flag leaf or second leaf was used to determine maximum quantum yield of photosystem II (PSII) photochemistry (F_v_/F_m_) using Pocket PEA (Hansatech Instruments, Ltd., Norfolk, UK). In total, 30 min was used for dark adaptation, and then a 1-s saturation flash was used to measure the potential maximum photochemical efficiency of PSII.

### 4.2. RNA-seq and Data Analysis

For the transcriptomic analysis, flag leaves of WT and TOSL3, the transgenic line with *OsNUC1-S* overexpression, were collected 3 and 9 days after the first day of the booting stage. Total RNA was extracted using Invitrogen’s Concert™ Plant RNA Reagent and then treated with DNaseI (NEB). A Dynabeads mRNA purification kit (Invitrogen, Carlsbad, CA, USA) and a KAPA Stranded mRNA-Seq Kit were used for mRNA isolation and cDNA libraries’ preparation, respectively. Fragment sizes of ~300 bp were selected and connected with adaptors. Then, the fragments were enriched by PCR for 12 cycles. All libraries were sequenced using the Genome Analyzer (Illumina HiSeq4000, San Diego, CA, USA). Adaptors were subsequently removed from all short-sequence reads before grouping, following the protocol of Missirian et al. [23]. All the sequences were aligned and mapped to the rice genome, and then, the differentially expressed genes (DEGs) were identified using the DESeq program [24]. The genes showing the differential expression were selected to validate with quantitative RT-PCR. *LOC_Os01g58470*, *LOC_Os01g64960*, *LOC_Os03g39610*, and *LOC_Os04g33830* predicted to localize in plastids were selected. The primers for detection of the gene expression are shown in Table 2. *OsEF-1∝* gene expression was used as an internal control. For q-RT-PCR, briefly, 1 μL of RNA extraction from either WT’s or transgenic rice’s flag leaves was used to synthesize the cDNA with an AccuPower^®^ RT PreMix (BIONEER, Oakland, CA, USA). Gene expression of the target genes was detected by quantitative PCR using Luna^®^ Universal qPCR Master MiX (BioLabs, San Diego, CA, USA). The thermal cycle was performed at 95 °C for 60 s, then 40 cycles of 95 °C for 15 s, 58.5 °C for 30 s, followed by 95 °C for 30 s, and the extension was done at 70 °C for 5 s. The relative expression of interesting genes was calculated using the Pfaffl method [25] with the formula:

Ratio = (E_target_) ^ΔCPtarget(control−sample)/^(E_ref_) ^ΔCPref(control−sample)^

### 4.3. Gene Ontology (GO) Term Analysis

Genes that had a *p*-value < 0.5 and log_2_fold change less than −1 or more than 1 were classified into GO terms using the ClueGO tool [26]. They were analyzed into three GO terms, cellular compartment, biological process, and molecular function, and subjected to a KEGG [27] pathway analysis.

### 4.4. Pigment Extraction and Quantification

Chlorophyll *a*, chlorophyll *b*, and total carotenoid contents were studied according to the method of Lichtenthaler [28]. Briefly, 50 mg fresh weight of either flag leaves or second leaves were extracted with 10 mL of 80% acetone and then incubated in the dark for 24 h at room temperature. Pigment extracts were measured at 470, 646.8, and 663.2 nm using a spectrophotometer (Agilent Technology, Santa Clara, CA, USA).

### 4.5. Yield Collection

After a 9 days salt-stress treatment, plants were recovered by the addition of water until soil salinity was below 2 ds·m^−1^. Seeds were harvested when they were fully developed and then desiccated. The numbers of panicles per plant and seeds per panicle, as well as the fertility rates (%), were determined.

### 4.6. Statistical Analysis

A randomized complete block design with four replications was used for the experimental plots. Analysis of variance was performed to detect the differences among means and a Tukey’s range test was used to detect significant differences between each mean at *p*-value < 0.05 using SPSS 21.0 statistical software. All the results are presented as mean ± standard error of the mean.

## 5. Conclusions

**Figure 11:**
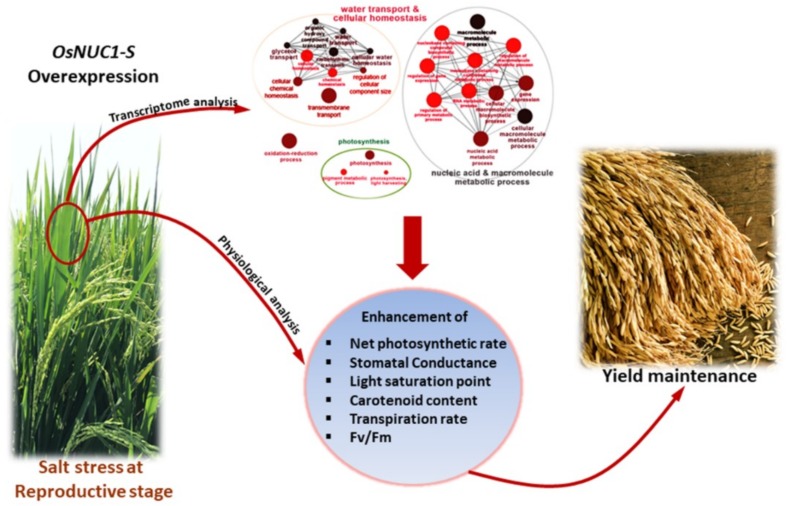
The scheme summarized the role of *OsNUC1-S* overexpression contributing to salt tolerance in rice.

Based on the experimental results, a role of *OsNUC1-S* in the salt tolerance of rice during the reproductive stage was suggested to be due to the enhancement of photosynthetic processes in both flag and second leaves through the modification of gene expression levels in water transport, photosynthesis, cellular homeostasis, and carotenoid biosynthesis. These could help maintain the grain yield after a salt stress (Figure 11).

## Figures and Tables

**Table 1:** Effects of salt stress at reproductive stage on tiller number per plant, panicle number per plant, panicle length, % fertility, and seed number per plant.

Reproductive Characters	Control *				Salt Stress *			
	WT	TOSL1	TOSL2	TOSL3	WT	TOSL1	TOSL2	TOSL3
Tiller number/pl	5.75 ± 0.55 ^ab^	5.25 ± 0.55 ^abc^	7.00 ± 0.55 ^a^	5.50 ± 0.55 ^abc^	3.00 ± 0.55 ^c^	3.75 ± 0.55 ^bc^	4.50 ± 0.55 ^abc^	4.00 ± 0.55 ^bc^
Panicle number/pl	5.25 ± 0.33 ^a^	3.50 ± 0.33 ^b^	3.50 ± 0.33 ^b^	3.00 ± 0.33 ^bc^	1.75 ± 0.33 ^c^	2.50 ± 0.33 ^bc^	2.75 ± 0.33 ^bc^	2.00 ± 0.33 ^bc^
Panicle length	10.15 ± 0.71	9.28 ± 0.71	8.87 ± 0.71	8.87 ± 0.71	7.38 ± 0.71	8.27 ± 0.71	9.00 ± 0.71	9.73 ± 0.71
% Fertility	65.36 ± 9.52 ^a^	45.18 ± 9.52 ^ab^	48.39 ± 9.52 ^ab^	44.34 ± 9.52 ^ab^	15.27 ± 9.52 ^b^	47.03 ± 9.52 ^ab^	14.17 ± 9.52 ^b^	36.84 ± 9.52 ^ab^
Seeds per plant	193.50 ± 11.15 ^a^	108.75 ± 11.15 ^bc^	130.00 ± 11.15 ^b^	115.75 ± 11.15 ^bc^	70.25 ± 11.15 ^c^	89.75 ± 11.15 ^bc^	68.50 ± 11.15 ^c^	84.50 ± 11.15 ^bc^

* The experiment was performed with random complete block design in four replicates, each of which consisted of two individuals. Analysis of variance was performed and means were compared with Tukey’s range test analysis. The data were presented as the mean ± SE and a different letter above the bar showed the significant difference in means (*p* < 0.05).

**Table 2:** Primers for gene expression detection.

Gene	Primer	Sequence (5′–3′)	Tm (°C)
*OsEF-1∝*	EF-1∝-F	ATGGTTGTGGAGACCTTC	53.7
	EF-1∝-R	ATGGTTGTGGAGACCTTC	58.2
*LOC_Os01g58470*	Os01g58470-F	AGGCATTGATCCTGAGACAG	54.3
	Os01g58470-R	AGAGCAGAATATCCCACTGC	54.4
*LOC_Os01g64960*	Os01g64960-F	GCATCGCCTTCTCCATCA	57.1
	Os01g64960-R	GAAGACGACGTTGAAGAGGA	57.3
*LOC_Os03g39610*	Os03g39610-F	GGAGGCGGTGTGGTTCAAGG	61.0
	Os03g39610-R	GCGGTAGCCCTCGACGAATC	60.3
*LOC_Os04g3383*	Os04g33830-F	CCGTTCTGGCTGTGGTT	55.4
	Os04g33830-R	CGTCCGTACAGTCAAGCTAA	54.3

## Supplementary Materials

The following are available online at http://www.mdpi.com/1422-0067/19/12/3936/s1.

## Abbreviations

Ci	intercellular CO_2_ concentration
F_v_/F_m_	intercellular CO_2_ concentration
g_s_	stomatal conductance
*OsNUC1*	rice *NUCLEOLIN1*
P_N_	net photosynthesis rate
WT	wild type
